# Gender differences in tuberculosis treatment outcomes: a post hoc analysis of the REMoxTB study

**DOI:** 10.1186/s12916-018-1169-5

**Published:** 2018-10-17

**Authors:** M. E. Murphy, G. H. Wills, S. Murthy, C. Louw, A. L. C. Bateson, R. D. Hunt, T. D. McHugh, A. J. Nunn, S. K. Meredith, C. M. Mendel, M. Spigelman, A. M. Crook, S. H. Gillespie, for the REMoxTB consortium, Andreas Diacon, Madeleine Hanekom, Amour Venter, Rodney Dawson, Kimberley Narunsky, B. Mtafya, N. Elias Ntinginya, Andrea Rachow, Evans Amukoye, B. Miheso, M. Njoroje, Noel Sam, D. Damas, Alphonce Liyoyo, A. Ahmad Mahayiddin, C. Chuchottaworn, J. Boonyasopun, B. Saipan, Shabir Lakhi, D. Chanda, J. Mcyeze, Alexander Pym, N. Ngcobo, Cheryl Louw, H. Veldsman, Gerardo Amaya-Tapia, T. Vejar Aguirre, D. K. Chauhan, R. K. Garg, N. K. Jain, A. Aggarwal, M. Mishra, S. Teotia, S. Charalambous, N. Hattidge, L. Pretorious, N. Padayachi, L. Mohapi, M. Gao, X. Li, L. Zhang, Q. Zhang, S. Aggarwal, Ketty Belizaire, Majda Benhayoun, D. Everitt, Ann Ginsberg, Martino Laurenzi, Bridget Rawls, Christopher Ridali, Mel Spigelman, Almarie Uys, Christo van Niekerk, Anna L. C. Bateson, Matthew Betteridge, S. Birkby, Emily Bongard, Michael Brown, Holly Ciesielczuk, C. Cook, E. Cunningham, James Huggett, Robert Hunt, Clare Ling, Marc Lipman, Paul Mee, Michael E. Murphy, Saraswathi E. Murthy, Felicity M. R. Perrin, Robert Shorten, Kasha P. Singh, K. Smith, Victoria Yorke-Edwards, Alimuddin Zumla

**Affiliations:** 1 UCL Centre for Clinical Microbiology, Division of Infection and Immunity, University College London, Royal Free Campus, Rowland Hill Street, London, NW3 2PF England, UK; 2 MRC Clinical Trials Unit at UCL, Institute for Clinical Trials and Methodology, Aviation House, 125 Kingsway, London, WC2B 6NH England, UK; 3 Madibeng Centre for Research, Brits, South Africa; 4 Department of Family Medicine, School of medicine, University of Pretoria, Pretoria, South Africa; 5 Global Alliance for Tuberculosis Drug Development, New York, NY 10005 USA; 6 School of Medicine, Medical and Biological Sciences Building, University of St Andrews, North Haugh, St Andrews, KY16 9TF Scotland, UK

**Keywords:** Gender, Tuberculosis, Treatment outcome, Cavitation, Clinical trials, REMoxTB

## Abstract

**Background:**

In the REMoxTB study of 4-month treatment-shortening regimens containing moxifloxacin compared to the standard 6-month regimen for tuberculosis, the proportion of unfavourable outcomes for women was similar in all study arms, but men had more frequent unfavourable outcomes (bacteriologically or clinically defined failure or relapse within 18 months after randomisation) on the shortened moxifloxacin-containing regimens. The reason for this gender disparity in treatment outcome is poorly understood.

**Methods:**

The gender differences in baseline variables were calculated, as was time to smear and culture conversion and Kaplan-Meier plots were constructed. In post hoc exploratory analyses, multivariable logistic regression modelling and an observed case analysis were used to explore factors associated with both gender and unfavourable treatment outcome.

**Results:**

The per-protocol population included 472/1548 (30%) women. Women were younger and had lower rates of cavitation, smoking and weight (all *p* < 0.05) and higher prevalence of HIV (10% vs 6%, *p* = 0.001). They received higher doses (mg/kg) than men of rifampicin, isoniazid, pyrazinamide and moxifloxacin (*p* ≤ 0.005). There was no difference in baseline smear grading or mycobacterial growth indicator tube (MGIT) time to positivity. Women converted to negative cultures more quickly than men on Lowenstein-Jensen (HR 1.14, *p* = 0.008) and MGIT media (HR 1.19, *p* < 0.001). In men, the presence of cavitation, positive HIV status, higher age, lower BMI and ‘ever smoked’ were independently associated with unfavourable treatment outcome. In women, only ‘ever smoked’ was independently associated with unfavourable treatment outcome. Only for cavitation was there a gender difference in treatment outcomes by regimen; their outcome in the 4-month arms was significantly poorer compared to the 6-month treatment arm (*p* < 0.001). Women, with or without cavities, and men without cavities had a similar outcome on all treatment arms (*p* = 0.218, 0.224 and 0.689 respectively). For all other covariate subgroups, there were no differences in treatment effects for men or women.

**Conclusions:**

Gender differences in TB treatment responses for the shorter regimens in the REMoxTB study may be explained by poor outcomes in men with cavitation on the moxifloxacin-containing regimens. We observed that women with cavities, or without, on the 4-month moxifloxacin regimens had similar outcomes to all patients on the standard 6-month treatment. The biological reasons for this difference are poorly understood and require further exploration.

**Electronic supplementary material:**

The online version of this article (10.1186/s12916-018-1169-5) contains supplementary material, which is available to authorized users.

## Background

Tuberculosis (TB) is amongst the leading causes of death in reproductive-age women. In 2014, there were 3.2 million incident TB cases in women and almost half a million deaths [1]. In pregnant women, TB is associated with significant increases in premature birth, low birth weight and death. In those co-infected with HIV, the risk of active TB is high, and there is a threefold risk of mother and child death [2].

While men are notified as having higher incident TB (5.4 million in 2015), and have higher mortality (16.5% vs 15%), there is a wide-ranging variation in gender differences geographically, and mortality is roughly equal in areas of highest HIV co-infection in Africa [1]. It is uncertain whether, in settings where there are low levels of HIV, women are biologically less susceptible to TB infection and reactivation or whether gender differences in TB incidence may reflect gender-specific sociocultural factors influencing TB exposure and/or access to healthcare [3, 4].

Most gender-specific TB research has focussed on differences in women’s access to healthcare and subsequent delays in seeking health services, with one study finding the status of being a married woman, a housewife or being a woman as being significantly associated with diagnostic delays [5]. There is evidence that women, once enrolled in healthcare, are more likely than men to adhere to the full course of treatment resulting in better treatment outcomes [6]. However, there are limited and sometimes conflicting data on gender differences in TB treatment responses and there may be specific factors, affecting either gender, influencing responses to treatment [7–9].

In the REMoxTB study of 4-month-treatment shortening regimens containing moxifloxacin, the proportion of favourable outcomes for women on the moxifloxacin-containing arms was similar to those on the standard 6-month treatment arm and would be considered non-inferior [10]. However, male patients, who comprised 70% of the study population, had significantly more unfavourable outcomes on the moxifloxacin-containing regimens. Specifically, while 8% of both males and females had unfavourable outcomes on the control regimen, male vs female breakdown of unfavourable rates for the isoniazid-moxifloxacin arm was 19% vs 7% and for the ethambutol-moxifloxacin regimen was 23% vs 13%. Although the study was not designed or powered to detect differences in treatment outcome by gender, the biological reasons for the observed gender disparity remain unclear and warrant further exploration.

The aim of this analysis was to better understand the biological and epidemiological factors associated with gender differences in TB treatment responses to inform future TB treatment and targeted public health interventions.

## Methods

We undertook an analysis of the REMoxTB study database [11]. Patients included in this secondary analysis were those in the pre-specified per-protocol population in whom the gender-by-treatment interaction was detected in the main REMoxTB study [10]. This was the primary analysis population for the trial including patients who had adhered to at least 80% of study drug.

### Patient treatment

Adult patients with sputum smear positive for acid-fast bacilli (AFB) were invited to be screened for enrolment to the REMoxTB study; a placebo-controlled, randomised, double-blind, phase 3 trial to test the non-inferiority of two experimental 4-month treatment arms containing moxifloxacin compared to standard 6-month treatment (see below). AFB-positive smears were confirmed on a new sputum sample in the study laboratory and additional blood and medical history were collected at the screening to determine patients’ eligibility, which are described elsewhere [10]. Patients with HIV infection could enrol with a CD4 > 250 cells/μL. Study sites were in Africa, Asia and Central America. Those eligible and consenting to enrolment in the study were randomised to receive the control regimen—2 months of rifampicin (R), isoniazid (H), ethambutol (E) and pyrazinamide (Z), followed by 4 months of rifampicin and isoniazid 2EHRZ/4HR —or one of the two experimental arms in which moxifloxacin (M) replaced either ethambutol (2MHRZ/2MHR; the isoniazid-arm) or isoniazid (2EMRZ/2MR; the ethambutol arm). Drug dosing was stratified by patient weight for rifampicin (< 45 kg, 450 mg; ≥ 45 kg, 600 mg), pyrazinamide (< 55 kg, 1000 mg; ≥ 55–75 kg, 1500 mg; > 75 kg, 2000 mg), and ethambutol (< 40 kg, 15 mg/kg rounded to nearest 100 mg; 40–55 kg, 800 mg; > 55–75 kg, 1200 mg; > 75 kg, 1600 mg), while patients received moxifloxacin 400 mg and isoniazid 300 mg, all according to their randomised allocated regimen.

### Microbiology

Patients enrolled in the REMoxTB study provided two sputum samples prior to commencing study drug. Further sputum samples were collected at regular study visits: weekly during the first 8 weeks of intensive phase treatment, at monthly visits until completion of study treatment at 6 months and three monthly for a further 12 months, with two samples being collected at each visit in the post-treatment phase. Each sputum sample was processed for smear microscopy and culture both on solid and in liquid media as per the REMoxTB-specific laboratory manual [12]. In brief, sputum samples were decontaminated and stained using Ziehl-Neelsen method and graded according to ATS guidelines as a semi-quantitative measure of mycobacterial burden [13]. Sputum samples were processed for culture on solid Lowenstein-Jensen (LJ) medium and in the fully automated BACTEC Mycobacterial Growth Indicator Tube system (MGIT; BBL™ MGIT™ 960, Becton Dickinson (BD) Microbiology Systems, Sparks, MD, USA). Time to detection (TTD) was recorded as a measure of mycobacterial burden. Drug susceptibility was performed on all isolates, and patients with multi-drug resistant TB, i.e. resistance to rifampicin ± isoniazid, were excluded.

### Statistical analyses

Clinical trial data were recorded in the study database along with patient demographics: gender, age, weight and individual drug dose per kilogramme, HIV status and smoking history. The extent of lung disease was quantified using a binary variable for cavitation (yes/no). In addition, Ralph et al. scoring was performed which provides a score out of 140 comprising percentage of lung involvement evident on chest radiograph with an additional 40 points for those with cavitation [14]. Treatment outcomes were as defined by the REMoxTB study in which the primary efficacy outcome was the proportion of patients who had bacteriologically or clinically defined failure or relapse within 18 months after randomisation (a composite unfavourable outcome). Differences in baseline characteristics, including mycobacterial burden, between males and females were compared using chi-squared (*χ*^2^) and Mann-Whitney *U* test. Kaplan-Meier plots were constructed to compare male and female time to smear and culture conversion, from randomisation to the study visit of the first negative result, summarised by a hazard ratio (HR) and compared using the logrank test. Factors found to be associated with gender (*p* < 0.1) were then included in a multivariable logistic regression model for unfavourable outcome, separately for men and women. Treatment effects within subgroups defined by covariates independently associated with outcome were explored, and an observed case analysis was conducted. All these analyses are post hoc and considered exploratory with no adjustments made for multiple testing. All analyses were conducted in Stata Version 14.0.

## Results

The per-protocol population of the REMoxTB study comprised 1548 patients, 472 (30%) of whom were female. Female patients were younger and had a higher BMI. They had lower rates of cavitation and smoking. Females received higher doses of rifampicin (11.28 mg/kg vs 10.99 mg/kg; *p* = 0.005), isoniazid (6.36 mg/kg vs 5.76 mg/kg; *p* = < 0.001), pyrazinamide (23.26 mg/kg vs 22.42 mg/kg; *p* < 0.001) and moxifloxacin (8.48 mg/kg vs 7.68 mg/kg; < 0.001), but not for ethambutol which had a higher dose in men (17.39 mg/kg vs 17.58 mg/kg; *p* = 0.018). Compared to males, females had higher prevalence of HIV (10% vs 6%; *p* = 0.001); but CD4 cell counts were comparable (437 and 405 cells/μL, *p* = 0.32) (see Table 1).

**Table 1:** Baseline characteristics stratified by gender and treatment group. Baseline characteristics of patients in the per-protocol population. Numbers are *N* (%) unless otherwise stated

Characteristics	Control group (*N* = 510)		Isoniazid group (*N* = 514)		Ethambutol group (*N* = 524)		All patients (*N* = 1548)	
Sex	Male (*N* = 356)	Female (*N* = 154)	Male (*N* = 351)	Female (*N* = 163)	Male (*N* = 369)	Female (*N* = 155)	Male (*N* = 1076)	Female (*N* = 472)
Age group*								
≤ 30 years	155 (44)	89 (58)	170 (48)	84 (52)	161 (44)	80 (52)	486 (45)	253 (54)
> 30 years	201 (56)	65 (42)	181 (52)	79 (48)	208 (56)	75 (48)	590 (55)	219 (46)
Weight group*								
< 40 kg	14 (4)	36 (23)	14 (4)	30 (18)	29 (8)	29 (19)	57 (5)	95 (20)
40–45 kg	45 (13)	35 (23)	54 (15)	36 (22)	54 (15)	28 (18)	153 (14)	99 (21)
> 45–55 kg	163 (46)	43 (28)	159 (45)	51 (31)	149 (40)	55 (35)	471 (44)	149 (32)
> 55 kg	134 (38)	40 (26)	124 (35)	46 (28)	137 (37)	43 (28)	395 (37)	129 (27)
BMI								
< 18.5	196 (55)	71 (46)	199 (57)	73 (45)	211 (57)	64 (41)	606 (56)	208 (44)
≥ 18.5	160 (45)	83 (54)	152 (43)	90 (55)	158 (43)	91 (59)	470 (44)	264 (56)
Race or ethnic group*^‡^								
Black	171 (48)	67 (44)	150 (43)	60 (37)	173 (47)	64 (41)	494 (46)	191 (40)
Asian	109 (31)	51 (33)	107 (30)	47 (29)	119 (32)	42 (27)	335 (31)	140 (30)
Mixed race or other	76 (21)	36 (23)	94 (27)	56 (34)	77 (21)	49 (32)	247 (23)	141 (30)
Smoking status*								
Never	133 (37)	113 (73)	121 (34)	110 (67)	127 (34)	103 (66)	381 (35)	326 (69)
Past	105 (29)	14 (9)	100 (28)	11 (7)	117 (32)	17 (11)	322 (30)	42 (9)
Current	118 (33)	27 (18)	130 (37)	42 (26)	125 (34)	35 (23)	373 (35)	104 (22)
HIV positivity*	21 (6)	17 (11)	23 (7)	14 (9)	17 (5)	18 (12)	61 (6)	49 (10)
Cavitation*^ǁ^	268 (75)	100 (65)	244 (70)	113 (69)	264 (72)	103 (66)	776 (72)	316 (67)
Area of lung involvement (%, (SD))	21.5 (12.5)	20.1 (12.4)	20.9 (13.1)	19.2 (12.1)	20.9 (12.2)	19.7 (11.0)	21.1 (12.6)	19.7 (11.9)
Smear grading								
Neg	14 (4)	7 (5)	14 (4)	5 (3)	17 (5)	7 (5)	45 (4)	19 (4)
1+	32 (9)	15 (10)	24 (7)	17 (10)	25 (7)	14 (9)	81 (8)	46 (10)
2+	43 (12)	19 (12)	52 (15)	26 (16)	62 (17)	22 (14)	157 (15)	67 (14)
3+	86 (24)	42 (27)	79 (23)	37 (23)	82 (22)	32 (21)	247 (23)	111 (24)
4+	181 (51)	71 (46)	182 (52)	78 (48)	183 (50)	80 (52)	546 (51)	229 (49)
LJ								
Positive	283 (79)	117 (76)	277 (79)	137 (84)	294 (80)	132 (85)	854 (81)	386 (82)
Negative	33 (9)	14 (9)	25 (7)	12 (7)	31 (8)	4 (3)	89 (8)	30 (6)
Contaminated	29 (8)	16 (10)	38 (11)	14 (9)	33 (9)	16 (10)	100 (9)	46 (10)
Indeterminate	11 (3)	7 (5)	11 (3)	0 (0)	11 (3)	3 (2)	33 (3)	10 (2)
TTD (median [IQR])	14 [14–21]	14 [14–21]	14 [14–25]	14 [14–21]	21 [14–28]	14 [14–21]	14 [14–21]	14 [14–21]
MGIT								
Positive	329 (92)	141 (92)	319 (93)	157 (96)	332 (90)	147 (95)	980 (93)	445 (94)
Negative	5 (1)	2 (1)	4 (1)	0 (0)	7 (2)	2 (1)	16 (2)	4 (1)
Contaminated	11 (3)	4 (3)	10 (3)	6 (4)	15 (4)	3 (2)	36 (3)	13 (3)
False positive	2 (1)	0 (0)	5 (1)	0 (0)	4 (1)	0 (0)	11 (1)	0 (0)
Indeterminate	9 (3)	7 (5)	13 (4)	0 (0)	11 (3)	3 (2)	13 (1)	10 (2)
TTP (median [IQR])	4.81 [3.71–6.66]	5.13 [3.79–6.79]	4.92 [3.70–6.39]	5.04 [4.03–6.43]	4.83 [3.67–6.58]	4.73 [3.70–6.50]	4.83 [3.71–6.50]	5.04 [3.88–6.46]

*p* values for categorical variables are calculated using chi-squared test and for the continuous variable using Mann-Whitney U test

*HIV* human immunodeficiency virus, *TTD* time to detect a positive culture in days on LJ media, *TTP* time to detect a positive culture in days in MGIT, *IQR* interquartile range

*Males and females were significantly different at baseline for weight, age, race, smoking, HIV, cavitation, area of lung involvement and LJ TTD; *p* values were ≤ 0.001, 0.002, 0.013, ≤ 0.001, 0.001, 0.022, 0.05 and 0.04 respectively

^‡^Race or ethnic group was reported by the investigator. Asian category included both South Asians and East Asians. Mixed race or other included mixed race, coloured and Caucasian

^ǁ^Cavitation status was missing for 148 patients

**Fig. 1:**
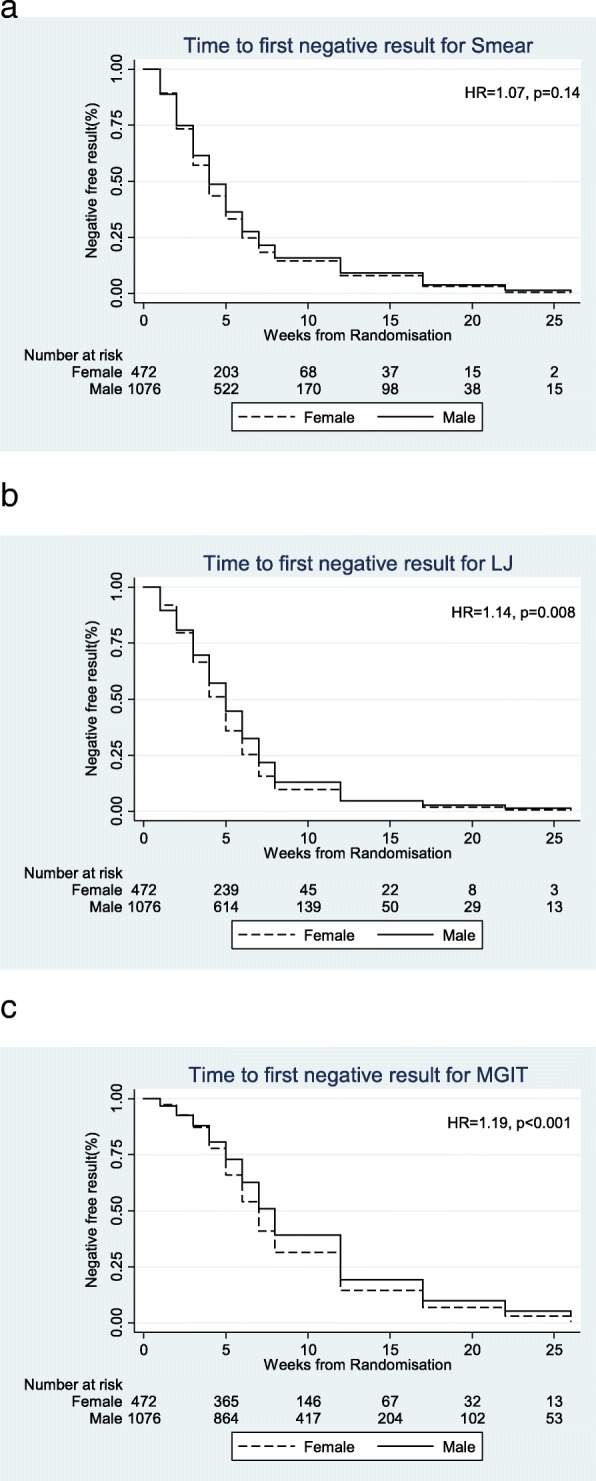
Kaplan-Meier estimates of time to **a** smear, **b** LJ and **c** MGIT culture conversion

There was no difference in pre-treatment smear gradings and MGIT time to positivity (TTP) (Table 1). The median LJ TTD was 14 days, with an interquartile range of 14–21 days, for both women and men, but there was a significant difference in their rankings with a lower LJ time to detection (TTD) suggesting higher mycobacterial burden in women (*p* = 0.04). Women were faster to convert to culture negative than men on both LJ (HR 1.14; 0.008) and in MGIT media (HR 1.19; *p* < 0.001). There was no difference in time to smear conversion (HR 1.07; *p* = 0.14). Kaplan-Meier plots are shown in Fig. 1.

Significant univariable baseline factors associated with an unfavourable outcome included cavitation (OR 2.19, *p* < 0.001), a current/ex-smoker (OR 2.07, *p* < 0.001), a low BMI (under 18.5 vs 18.5 and above) (OR 1.53, *p* = 0.004), being older (30 years and under vs over 30 years) (OR 1.6, *p* = 0.003) and HIV (OR 2.26, *p* < 0.001) and were included in the final adjusted logistic regression models for unfavourable outcome including treatment arm, for men and women separately. Drug dosing and baseline smear and culture results were not significantly associated with unfavourable outcome and were not included in the model. Ralph scoring, which includes a measure of the percentage of lung involvement in addition to the binary cavity variable, showed borderline evidence that men had a higher overall percentage of lung involvement than women (21.1% vs 19.7%; *p* = 0.05), but inclusion of this variable in the model did not improve the overall fit of the models when considered in place of the binary cavitation variable. Results are shown in Tables 2 and 3. For men, all factors included remained significantly associated with unfavourable outcome in multivariable analysis, except for race. For women, only current/ex-smoking status was significantly associated with the unfavourable response. No significant treatment-by-covariate interactions were observed in the multivariable models, which might have been expected given the small numbers in each subgroup.

**Table 2:** Treatment and covariate effects on unfavourable outcome for men (*N* = 974)

	r/n	OR (unadjusted), 95% CI	*p*	aOR (adjusted), 95% CI	*p*
Treatment					
HRZE	30/326 (33)			1.0	
2MHRZ/2MHR	55/312 (32)	2.22 (1.36–3.62)	0.001	2.24 (1.37–3.65)	0.001
2EMRZ/2MR	78/336 (35)	3.31 (2.07–5.29)	< 0.001	3.31 (2.07–5.28)	< 0.001
Age					
≤ 30 years	58/435	1.0		1.0	
> 30 years	105/539	1.46 (1.00–2.12)	0.045	1.48 (1.02–2.14)	0.037
BMI					
< 18.5	105/547	1.0		1.0	
≥ 18.5	58/427	0.65 (0.45–0.93)	0.020	0.64 (0.44–0.92)	0.017
Race					
Asian	50/311	1.0			
Black	70/430	0.85 (0.55–1.32)	0.469	–	–
Mixed race and Caucasian	43/233	0.97 (0.58–1.61)	0.902		
Smoking					
Never smoked	39/334	1.0		1.0	
Ever smoked	124/640	1.61 (1.04–2.49)	0.034	1.60 (1.07–2.40)	0.023
HIV					
Negative	143/922 (95)	1.0		1.0	
Positive	20/52	4.26 (2.22–8.18)	< 0.001	3.97 (2.12–7.42)	< 0.001
Cavities					
No cavities	16/198	1.0		1.0	
Cavities	147/776	2.78 (1.59–4.85)	< 0.001	2.78 (1.59–4.84)	< 0.001

*r* number with unfavourable outcome

*n* total in category

**Table 3:** Treatment and covariate effects on unfavourable outcome for women (*N* = 426)

	r/n	OR (unadjusted), 95% CI	*p*	aOR (adjusted), 95% CI	*p*
Treatment					
HRZE	10/138				
2MHRZ/2MHR	11/149	0.98 (0.40–2.43)	0.973	–	–
2EMRZ/2MR	18/139	1.93 (0.84–4.39)	0.119		
Age					
≤ 30 years	20/223	1.0		–	–
> 30 years	19/203	0.95 (0.48–1.88)	0.881		
BMI					
< 18.5	19/189	1.0		–	–
≥ 18.5	20/237	1.03 (0.51–2.12)	0.926		
Race					
Asian	15/135	1.0			
Black	9/163	0.39 (0.14–1.08)	0.070	–	–
Mixed race and Caucasian	15/128	0.45 (0.14–1.45)	0.182		
Smoking					
Never smoked	21/292	1.0		1.0	
Ever smoked	18/134	2.69 (0.92–7.90)	0.071	2.00 (1.03–3.90)	0.041
HIV					
Negative	36/385	1.0		–	–
Positive	3/41	1.16 (0.28–4.84)	0.835		
Cavities					
No cavities	9/110	1.0		–	–
Cavities	30/316	1.16 (0.51–2.63)	0.715		

In exploring the treatment effects within subgroups descriptively, cavitation emerged as the only covariate whose statistical significance differed substantially between genders in terms of treatment outcomes by regimen. Men with cavities had significantly poorer outcomes compared to women with cavities (19% vs 9%, *p* < 0.001; Table 4). In contrast, men and women without cavitation had similar treatment outcomes (both 9%, *p* = 0.975). Men with cavities had worse treatment outcomes than men without cavities and had significantly poorer outcomes on the experimental arms compared to control (*p* < 0.001; Table 5). However, women with cavities had no difference in treatment outcome compared to women without cavities (Table 4), and women, with or without cavities, and men without cavities had similar treatment outcomes regardless of the treatment regimen (*p* = 0.218, 0.224 and 0.689 respectively).

**Table 4:** Unfavourable outcome within gender and subgroups defined by cavities

Cavities (*N* = 1092)				No cavities (*N* = 308)			
Male *N* (%)		Female *N* (%)		Male *N* (%)		Female *N* (%)	
776 (71)		316 (29)		198 (64)		110 (36)	
Favourable	Un-fav	Favourable	Un-fav	Favourable	Un-fav	Favourable	Un-fav
629 (81)	147 (19)	286 (91)	30 (9)	182 (92)	16 (8)	101 (92)	9 (8)
*p* < 0.001				*p* = 0.975

**Table 5:** Unfavourable outcome by treatment group within gender and subgroups defined by cavities

	2EHRZ/4HR	2MHRZ/2MHR	2EMRZ/2MR
Men with cavities	268	244	264
Favourable	241 (90)	193 (79)	195 (74)
Unfavourable	27 (10)	51 (21)	69 (26)
*p* < 0.001			
Men without cavities	58	68	72
Favourable	55 (95)	64 (94)	63 (88)
Unfavourable	3 (5)	4 (6)	9 (13)
*p* = 0.224			
Women with cavities	100	113	103
Favourable	93 (93)	104 (92)	89 (86)
Unfavourable	7 (7)	9 (8)	14 (14)
*p* = 0.218			
Women without cavities	38	36	36
Favourable	35 (92)	34 (94)	32 (89)
Unfavourable	3 (8)	2 (6)	4 (11)
*p* = 0.689			

To ensure that this result is not impacted by missing cavitation results, we repeated these analyses using imputed values (*N* = 148) employing a multiple imputation approach, and this produced similar results (data not shown). No other treatment effect differences across covariate subgroups within gender including HIV status, smoking and BMI were observed to explain the gender-by-treatment interaction found in the REMoxTB study (see Additional file 1: Tables S1).

## Discussion

Women receiving 4-month moxifloxacin-containing regimens in the REMoxTB study had similar outcomes to those on 6-month control regimens. This held true for all covariate subgroups of women including HIV, smoking and low BMI and seems biologically plausible as women responded faster to TB treatment than men, despite comparable pre-treatment mycobacterial burdens. In contrast, the 4-month regimens, men had a significantly worse outcome compared to standard 6-month therapy, particularly the ethambutol-containing regimen. Cavitation was the only baseline characteristic measured which could potentially explain the observed difference in treatment outcomes between men and women.

Our analyses show that failure in the REMoxTB study was driven by poorer outcomes in men with cavitation in the moxifloxacin-containing arms. Importantly, men without cavities and women, with or without cavities, had similar outcomes in the moxifloxacin-containing and standard regimens. In addition to cavitation, men had poorer treatment outcomes on the experimental arms compared to control in all other covariate subgroups. While age, BMI, smoking status and HIV status were associated with an unfavourable outcome in males, they could not explain the different outcome in the individual treatment regimens. Similarly, for women, a history of smoking increased the hazard of a poor outcome, but there was no difference in outcomes across treatment regimens.

It is already established in a previous paper from our group that cavities visible on posterior-anterior chest radiograph are associated with the mycobacterial load as measured by time to positivity (TTP) and directly related to the size of the cavity [15]. The rate of decline of mycobacterial burden in that paper was unrelated to baseline load suggesting that patients with higher mycobacterial burdens at baseline would take longer to culture convert. However, in the current study, while the poor outcomes of men with cavitation were the only factor which may in part possibly explain the gender-by-treatment interaction, males and females had comparable mycobacterial burdens as measured by MGIT TTP prior to starting the treatment, yet women were faster to culture convert. The significant difference in the mycobacterial burden on LJ was more likely related to the ranking of categorical TTD data, recorded weekly than any real difference. In any case, this suggested a higher mycobacterial burden in women and would therefore have been expected to favour males. Furthermore, although we did not measure cavity volume specifically, and while there was borderline evidence of a higher percentage of lung involvement for men compared to women, this additional information did not improve the fit of our statistical model.

Cavitation has previously been identified as a risk factor for poor outcomes in TB treatment regimens, but these have not been stratified by gender [16]. The poor outcome of males with cavities on the experimental regimens compared to females with cavities cannot easily be explained. It may be that males had a higher volume of cavities, which is a factor that is not measured by the Ralph score, as this measure includes a single binary ‘penalty’ for cavities that is added to the score for percentage lung involvement. Studies of TB immunopathology have identified matrix-metalloproteinases as crucial factors controlling the pulmonary extracellular matrix involved in cavity formation [17]. A recent study of the collagenase MMP-8 in plasma has shown this to be higher in males than in females which may support greater cavitation in male patients and deserves further consideration, along with other potential gender-specific immunological factors which might explain the findings of this study [18].

An earlier randomised control trial comparing 4-month and 6-month standard regimens in 394 patients, including 154 women, with non-cavitary disease, and who culture converted after 2 months standard treatment, was halted due to an unacceptable failure rate in the 4-month arms (7.0% vs 1.6%). This suggests that cavitation may not entirely explain the gender difference in treatment outcome observed in the REMoxTB study, however, again, the results of this study were not reported by gender [19]. A re-analysis of previous trial data from the UK MRC comparing 4- and 6-month regimens also identified higher rates of failure in the shorter regimens (5.9% vs 0%) [20]. However, unpublished data from two previous MRC trials involving unsuccessful 4-month regimens, including one containing moxifloxacin, indicated that women had significantly better outcomes than men in an analysis stratified by cavitation, as in our study (Personal communication: Professor Andrew Nunn, MRC Clinical Trials Unit at UCL).

Gender-specific pharmacodynamics might potentially explain the observed differences in the treatment outcome. In the REMoxTB study, women, on average, received small increased doses of four of five study drugs, including moxifloxacin, known to be essential for bacterial sterilisation and cure. Increased dosing may therefore go some way to explaining the faster bacteriological response to treatment, but these were not found to be significantly associated with treatment outcome on univariable analyses. No gender difference in the pharmacokinetics of moxifloxacin has been described to explain the differences in unfavourable outcome between men and women with and without cavitation on the moxifloxacin-containing regimens. Poor outcomes on the ethambutol arms may be due to the superior bactericidal effect of isoniazid or the presence of three drugs over a 4-month period. Compliance with study regimens may also be a factor. To be included in the per-protocol analysis in which the gender-by-treatment interaction was identified, all patients had to have taken more than 80% of their medication. However, as data collection was not sufficiently detailed to address adherence further by gender, we do not know whether, within the per-protocol population of the REMoxTB study taking >80% of study medication, females may have had significantly greater compliance, nearer 100%, compared to men, or, indeed, vice versa. A previous systematic review of previous studies found a higher likelihood of compliance amongst females, so it would be important to consider the potential impact in future studies [6]. Furthermore, we were unable to further stratify compliance by gender and cavitation, and we cannot comment on whether there were differences in treatment compliance in men and women with cavitation which might explain the differences observed in the REMoxTB study.

To date, reports of gender differences in outcome have often been excluded from published clinical trials of moxifloxacin, and thus, there is limited data on the outcomes by gender for the many indications of moxifloxacin [21]. Two other clinical trials of fluoroquinolones for tuberculosis were published at the same time as the REMoxTB study but neither included analysis by gender [22, 23]. The US Food and Drug Administration, guidelines support reporting of gender differences in the clinical evaluation of drugs and journals are increasingly introducing editorial policies requiring the reporting of result by gender [24, 25]. Our observation emphasises the importance of such policies and supports the reporting of outcomes by gender so that we can better understand the factors bearing on these differences. This is particularly true for studies of moxifloxacin given that it is commonly used to treat with complicated and/or severe disease including patients intolerant of other first-line drugs and in patients with TB meningitis.

Although we should bear in mind that these analyses were all post hoc, considered exploratory and based on relatively small numbers not powered to detect a gender-treatment interaction, the findings suggest that possibly the shorter regimens may be appropriate in females. Yet, how gender-specific therapy, if indicated, could be implemented within current standard National TB Programmes requires operational consideration. Research on gender difference in tuberculosis has thus far focussed on improving access to healthcare for women, presuming that, once engaged, women will have greater adherence to therapy. It is axiomatic that we need to improve patient engagement and adherence to approved regimens, but our study suggests a greater focus on men may be required to improve their treatment outcomes. This is supported by the findings of a recent meta-analysis that reported men as disadvantaged in seeking or accessing TB services and suggested that men were a high-risk group requiring improved access to TB [4]. Other factors associated with poor outcomes in men and/or women, including smoking and HIV, should further assist in directing public health responses.

## Conclusions

Gender differences in TB treatment responses for the shorter regimens in the REMoxTB study may be explained by poor outcomes in men with cavitation on the moxifloxacin-containing regimens. We observed that women with cavities, or without, on the 4-month moxifloxacin regimens had similar outcomes to all patients on the standard 6-month treatment. The biological reasons for this difference are poorly understood and require further exploration.

## Additional files

Additional file 1: **Table S1.** a. Outcome by treatment within gender and HIV subgroups. b. Outcome by treatment within gender and smoker subgroups. (DOCX 20 kb)

Additional file 2: List of ethics committee approving the REMoxTB study. (DOCX 892 kb)

## Abbreviations

AFB: Acid-fast bacilli
ATS: American Thoracic Society
BMI: Body mass index
E: Ethambutol
H: Isoniazid
HIV: Human immunodeficiency virus
HR: Hazard ratio
IQR: Interquartile range
LJ: Lowenstein-Jensen
M: Moxifloxacin
MGIT: Mycobacterial growth indicator tube
MMP: Matrix metalloproteinase
MRC: Medical Research Council
Neg: Negative
OR: Odds ratio
R: Rifampicin
REMoXTB: Rapid regulatory evaluation of moxifloxacin for TB
TB: Tuberculosis
TTD: Time to detection
TTP: Time to positivity
UCL: University College London
UK: United Kingdom
Un-fav: Unfavourable (outcome)
US: United States
WHO: World Health Organization
Z: Pyrazinamide
ZN: Ziehl-Neelsen
