# Supplementary material for: Gender differences in tuberculosis treatment outcomes: a post hoc analysis of the REMoxTB study
Source: BMC Med. 2018 Oct 17;16:189. doi: 10.1186/s12916-018-1169-5 (PMC6192317; doi:10.1186/s12916-018-1169-5)
Supplement: Supplementary file 1 — Table S1. a. Outcome by treatment within gender and HIV subgroups. b. Outcome by treatment within gender and smoker subgroups. (DOCX 20 kb) [file 12916_2018_1169_MOESM1_ESM.docx]

Supplementary tables showing unfavourable outcomes by gender and subgroups defined by covariates.

| **Table S1a. Outcome by treatment within gender and HIV subgroups** | | | |
| --- | --- | --- | --- |
|  | 2EHRZ/4HR | 2MHRZ/2MHR | 2EMRZ/2MR |
| ***Men with HIV*** | 19 | 19 | 14 |
| Favourable | 14 (74) | 13 (68) | 5 (36) |
| Unfavourable | **5 (26)** | **6 (32)** | **9 (64)** |
| **P=0.064** | | | |
| ***Men without HIV*** | 307 | 293 | 322 |
| Favourable | 282 (92) | 244 (83) | 253 (79) |
| Unfavourable | **25 (8)** | **49 (17)** | **69 (21)** |
| **P<0.001** | | | |
| ***Women with HIV*** | 15 | 13 | 13 |
| Favourable | 15 (100) | 12 (92) | 11 (85) |
| Unfavourable | **0** | **1 (8)** | **2 (15)** |
| **p=0.296** | | | |
| ***Women without HIV*** | 123 | 136 | 126 |
| Favourable | 113 (92) | 126 (93) | 110 (87) |
| Unfavourable | **10 (8)** | **10 (7)** | **16 (13)** |
| **p=0.283** | | | |

| **Table S1b. Outcome by treatment within gender and smoker subgroups** | | | |
| --- | --- | --- | --- |
|  | 2EHRZ/4HR | 2MHRZ/2MHR | 2EMRZ/2MR |
| ***Men who have smoked*** | 204 | 209 | 227 |
| Favourable | 181 (89) | 164 (78) | 171 (75) |
| Unfavourable | **23 (11)** | **45 (22)** | **56 (25)** |
| **P=0.001** | | | |
| ***Men who have not smoked*** | 122 | 103 | 109 |
| Favourable | 115 (94) | 93 (90) | 87 (80) |
| Unfavourable | **7 (6)** | **10 (10)** | **22 (20)** |
| **p=0.002** | | | |
| ***Women who have smoked*** | 39 | 50 | 45 |
| Favourable | 34 (87) | 44 (88) | 38 (84) |
| Unfavourable | **5 (13)** | **6 (12)** | **7 (16)** |
| **p=0.871** | | | |
| ***Women who have not smoked*** | 99 | 99 | 94 |
| Favourable | 94 (95) | 94 (95) | 83 (88) |
| Unfavourable | **5 (5)** | **5 (5)** | **11 (12)** |
| **p=0.121** | | | |

| **Table S1c. Outcome by treatment within gender and BMI subgroups** | | | |
| --- | --- | --- | --- |
|  | 2EHRZ/4HR | 2MHRZ/2MHR | 2EMRZ/2MR |
| ***Men who are 18.5 and over*** | 144 | 137 | 146 |
| Favourable | 131 (91) | 118 (86) | 120 (82) |
| Unfavourable | **13 (9)** | **19 (14)** | **26 (18)** |
| **P=0.092** | | | |
| ***Men who are under 18.5*** | 182 | 175 | 190 |
| Favourable | 165 (91) | 139 (79) | 138 (73) |
| Unfavourable | **17 (9)** | **36 (21)** | **52 (27)** |
| **P<0.001** | | | |
| ***Women who are 18.5 and over*** | 75 | 82 | 80 |
| Favourable | 69 (92) | 78 (95) | 70 (88) |
| Unfavourable | **6 (8)** | **4 (5)** | **10 (13)** |
| **p=0.215** | | | |
| ***Women who are under 18.5*** | 63 | 67 | 59 |
| Favourable | 59 (94) | 60 (90) | 51 (86) |
| Unfavourable | **4 (6)** | **7 (10)** | **8 (14)** |
| **p=0.413** | | | |
