# Supplementary figures and images for: Gender differences in tuberculosis treatment outcomes: a post hoc analysis of the REMoxTB study
Source: BMC Med. 2018 Oct 17;16:189. doi: 10.1186/s12916-018-1169-5 (PMC6192317; doi:10.1186/s12916-018-1169-5)

Supplement S2 List of ethics committee approving the REMoxTB study


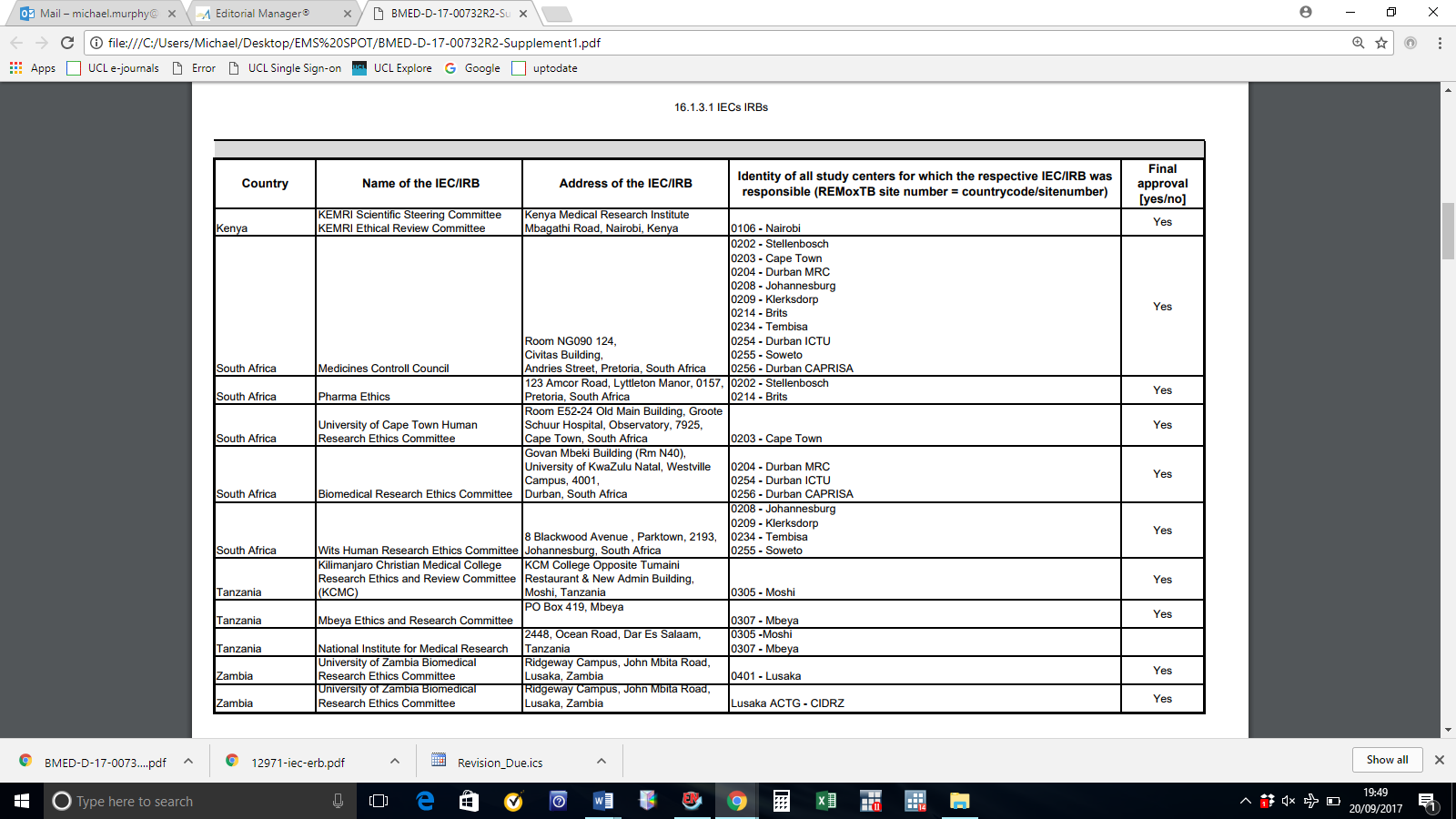


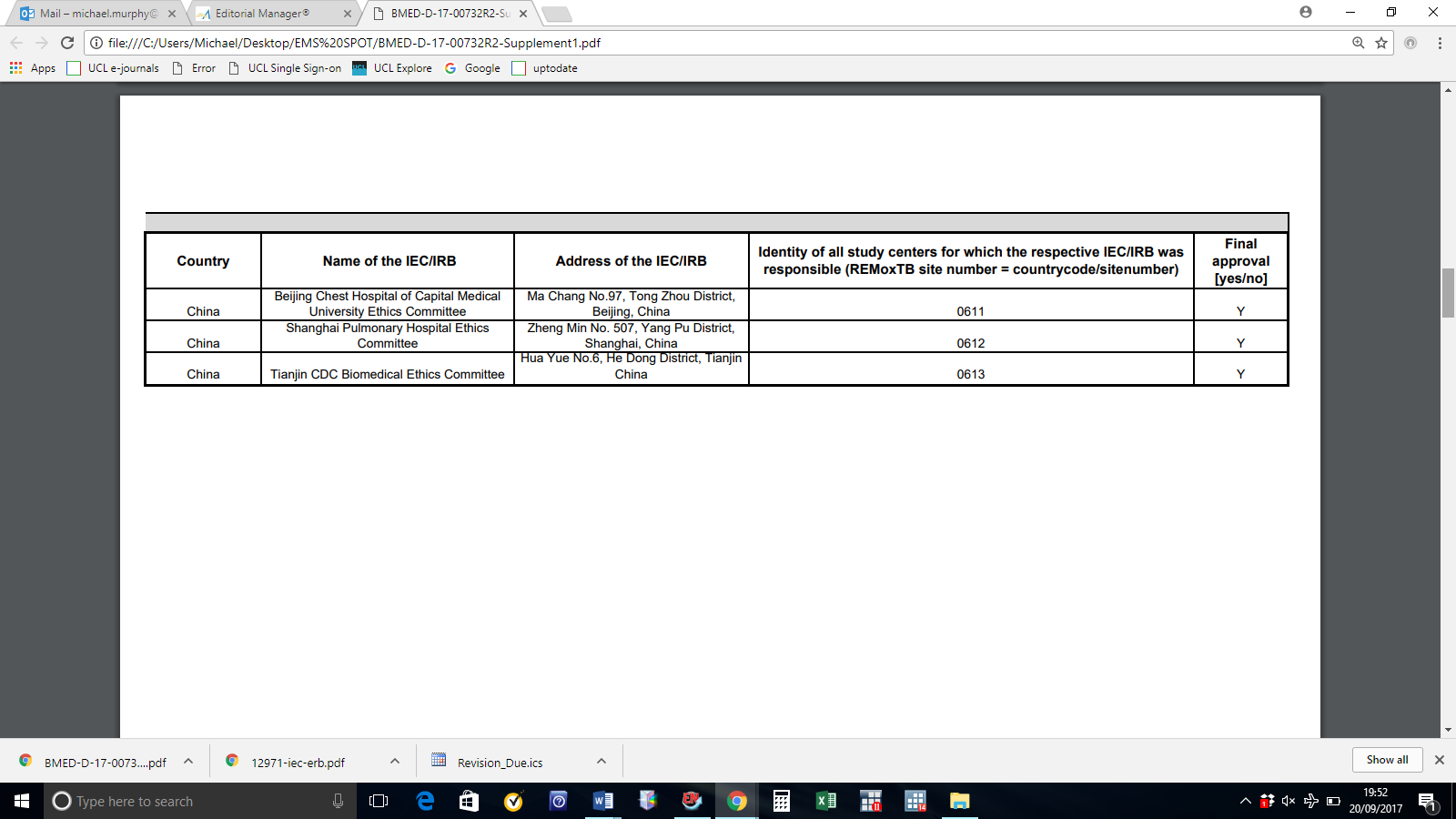


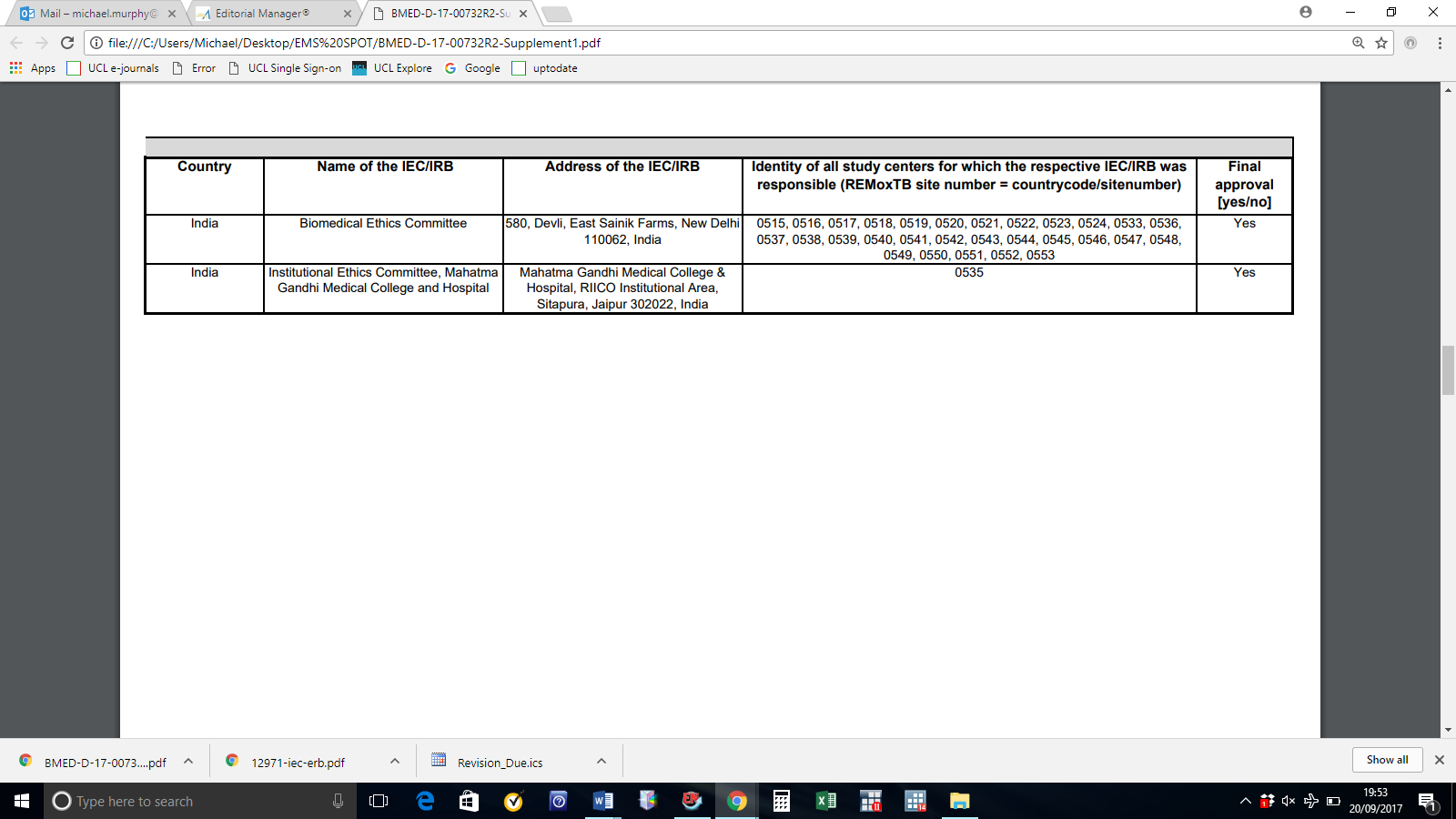


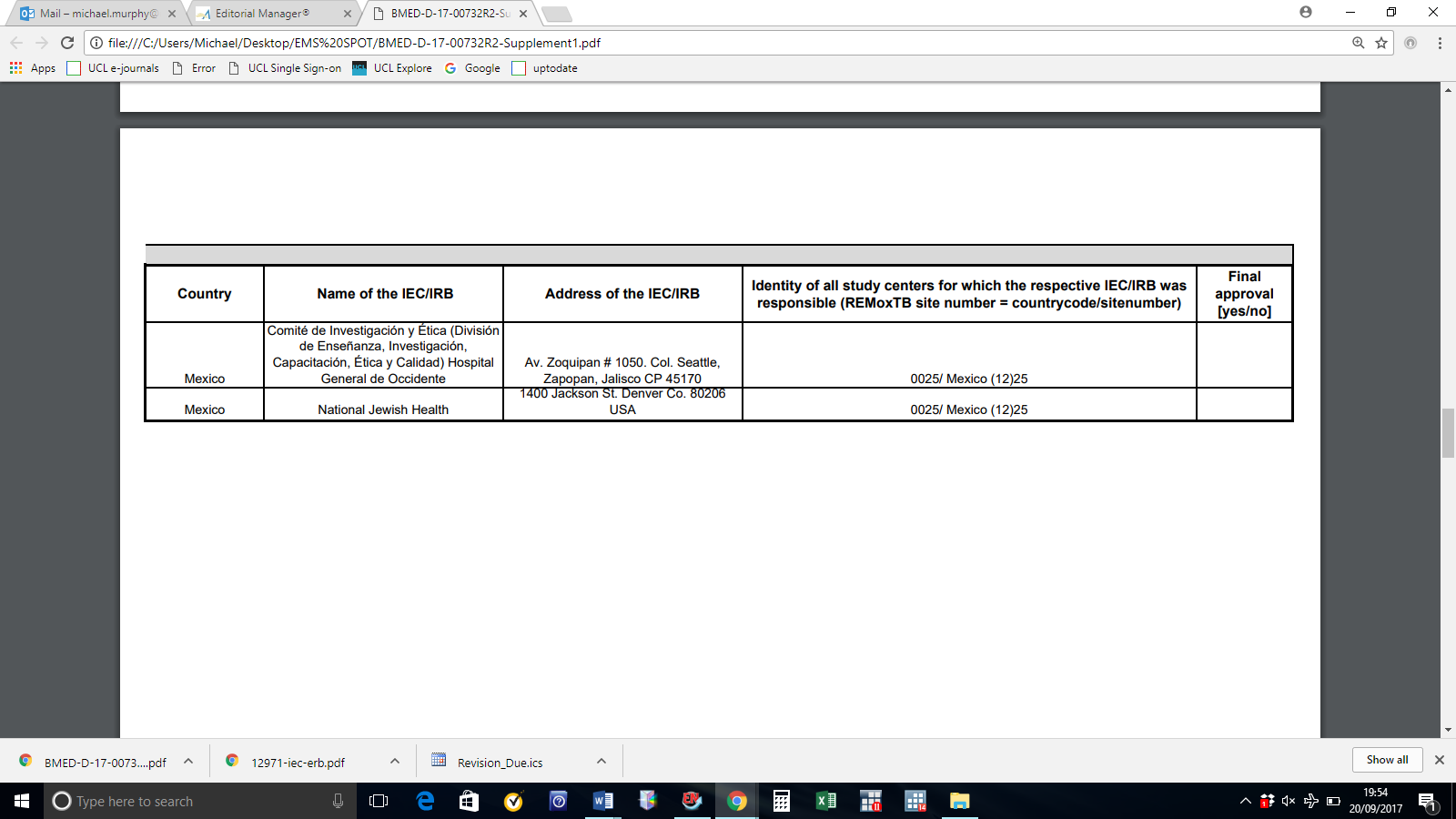


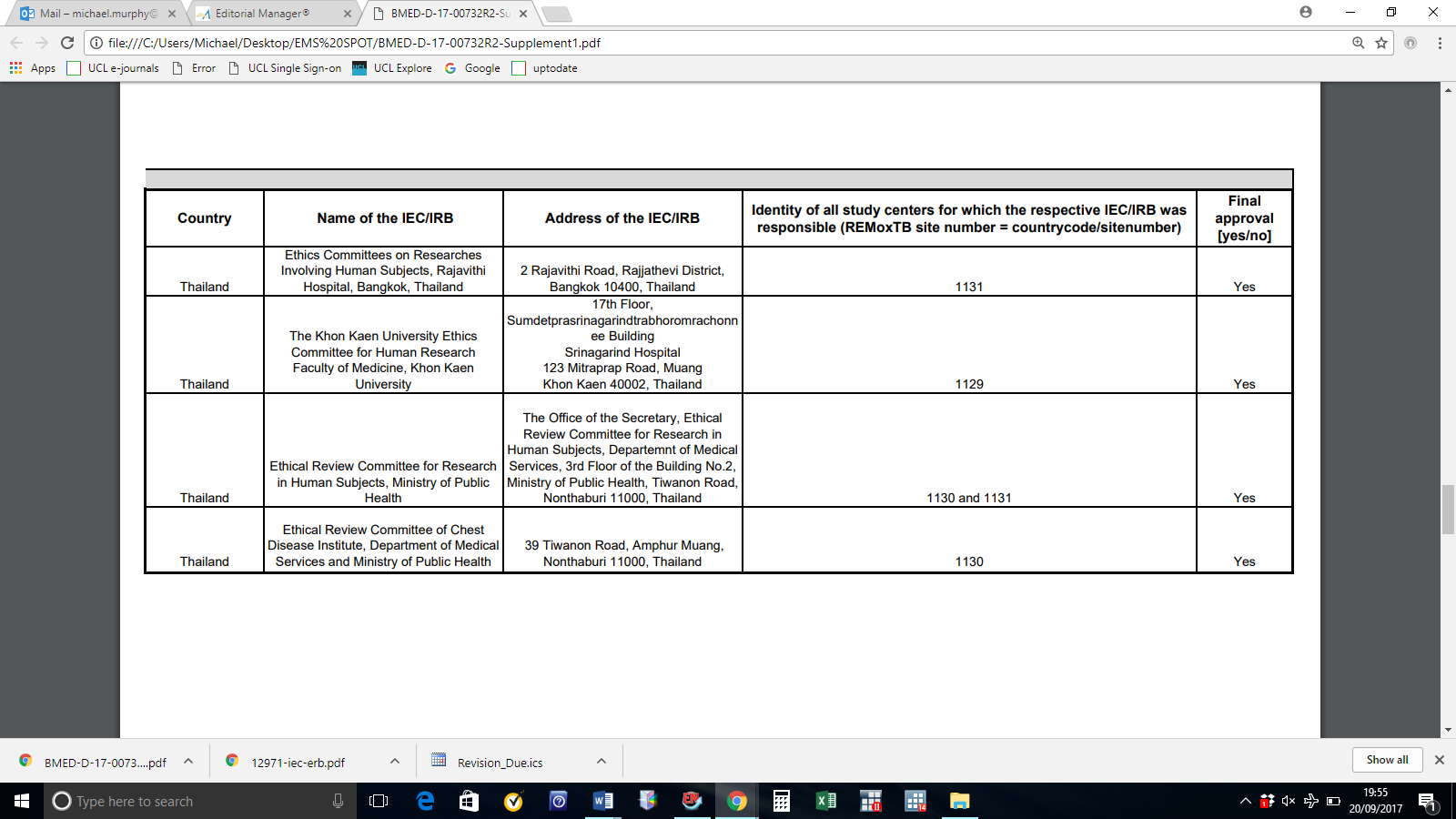


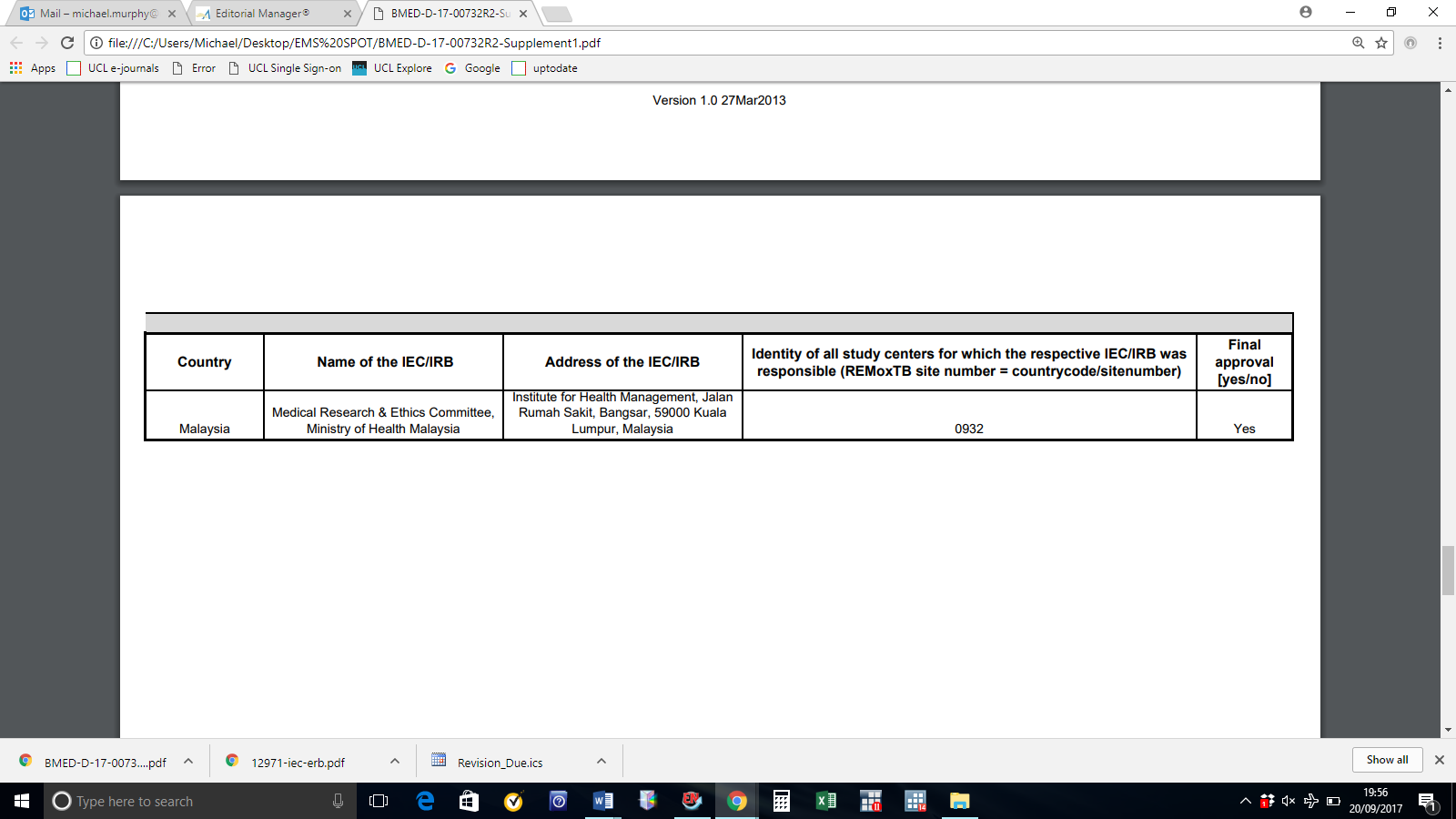

Supplement: Supplementary file 2 — List of ethics committee approving the REMoxTB study. (DOCX 892 kb) [file 12916_2018_1169_MOESM2_ESM.docx]
